# Purification and characterization of a novel defensin from the salivary glands of the black fly, *Simulium bannaense*

**DOI:** 10.1186/s13071-015-0669-9

**Published:** 2015-02-04

**Authors:** Lin Wei, Lixian Mu, Yipeng Wang, Hui Bian, Jun Li, Yiling Lu, Yi Han, Tong Liu, Jing Lv, Cuiping Feng, Jing Wu, Hailong Yang

**Affiliations:** School of Basic Medical Sciences, Kunming Medical University, Kunming, Yunnan China; Jiangsu Key Laboratory of Infection and Immunity, Institute of Biology and Medical Sciences, Soochow University, Suzhou, Jiangsu China; College of Pharmaceutical Sciences, Soochow University, Suzhou, Jiangsu China; Institute of Marine biological technology, School of Life Science and Biotechnology, Dalian University of Technology, Dalian, Liaoning China

**Keywords:** Insect, Antimicrobial peptide, Defensin, Salivary gland, Black fly, *Simulium bannaense*

## Abstract

**Background:**

Black flies (Diptera: Simuliidae) are haematophagous insects that can cause allergic reactions and act as vectors of pathogens. Although their saliva has been thought to contain a diverse array of physiologically active molecules, little information is available on antimicrobial factors in black fly salivary glands, especially no defensins have been reported so far.

**Methods:**

A novel cationic defensin designated *Siba*Def was purified using reverse phase high-performance liquid chromatography (RP-HPLC) from the salivary glands of the black fly *Simulium bannaense*. The amino acid sequence of *Siba*Def was determined by a combination method of automated Edman degradation and cDNA sequencing. The morphologic changes of Gram-positive bacteria *Staphylococcus aureus* or *Bacillus subtilis* treated with *Siba*Def were assessed by scanning electron microscopy (SEM). Quantitative PCR (qPCR) was performed to analyze the expression of *Siba*Def mRNA in whole bodies of insects after oral infection with the bacteria *S. aureus* or *B. subtilis*.

**Results:**

Surprisingly, the phylogenetic analysis of defensin-related amino acid sequences demonstrated that *Siba*Def is most closely related to defensins from the human body louse *Pediculus humanus corporis* (Anoplura: Pediculidae), rather than to other dipteran defensins. *Siba*Def showed potent antimicrobial activities against Gram-positive bacteria with minimal inhibitory concentrations (MICs) ranging from 0.83 μM to 2.29 μM. SEM analysis indicated that *Siba*Def killed microorganisms through the disruption of cell membrane integrity. The transcript levels of *Siba*Def in the bacteria-immunized flies increased with the time course, reaching maximum at 36 h and then slowly decreased from that time point.

**Conclusions:**

Our results indicate that *Siba*Def is involved in the innate humoral response of the black fly *S. bannaense*, and it might play a significant role in the defence against microorganisms in both sugar and blood meals.

## Background

Black flies (Diptera: Simuliidae) are closely related to some blood-sucking insects such as mosquitoes and biting midges [1,2]. They are not only a biting nuisance for humans and livestock but also transmit diseases including human onchocerciasis (river blindness) caused by the nematode *Onchocerca volvulus* and livestock disease caused by vesicular stomatitis virus [3-5]. To facilitate a blood meal, haematophagous Diptera have developed an extraordinary array of salivary proteins that can overcome the host’s hemostatic barriers, as well as suppressing inflammatory and immunologic reactions [6-9]. In addition, these Diptera also take sugar meals.

Several salivary anti-haemostatic factors have been identified from black fly, including inhibitors of coagulation factors (Factor Xa, V and thrombin), potent vasodilators (*Simulium vittatum* erythema proteins, SVEPs) and anti-platelet aggregation factors (apyrase) [10-16]. Hyaluronidase and immunomodulatory activities have also been described in *S. vittatum* salivary gland extract [5,17,18].

As an important hematophagous arthropod, there was not much information available about pharmacologically active compounds in black fly salivary glands, until salivary transcriptomes have been made and described from three black fly species (*S. guianense, S. vittatum* and *S. nigrimanum*) [19-21]. In these studies, many more substances with potential anti-haemostatic functions or immunity-related activities have been uncovered. Immunity-related gene products including six antimicrobial peptides of the cecropin family, nine lysozymes, and three members of the Gram-negative bacteria-binding protein, have been identified from these three species. However, no antimicrobial peptide (AMP) belonging to the defensin family has so far been biochemically characterized from black fly.

Insect defensins are a class of gene-encoded effector molecules of innate immunity. They have six strictly conserved cysteine residues linked in the 1–4, 2–5, 3–6 pattern, except for the antifungal peptide drosomycin from *Drosophila melanogaster*, which has eight cysteine residues forming four stabilizing disulfide bridges [22]. So far, more than 60 defensins have been identified from different species of insect orders (Diptera, Lepidoptera, Hymenoptera, Hemiptera, Isoptera, Coleoptera and Odonata) [22,23], The majority of insect defensins were isolated from the haemolymph, fat body or midgut of bacteria-immunized larvae [24-27], while such defensins were seldom reported from the saliva or salivary glands. In the current work, we firstly report the purification and characterization of the defensin from the black fly salivary glands.

## Methods

### Black fly salivary gland dissection

Adult *S. bannaense* (about 2,000 flies) were collected near streams in Xishuangbanna, Yunnan, China (21.556°N 101.162°E). The collections were made in five months (April-May, September-October 2013; May 2014). The black fly salivary glands (1,800 pairs) used for protein extraction (1,660 pairs) or total RNA extraction (140 pairs), were dissected in ice cold HEPES saline (10 mM HEPES pH 7.2, 150 mM NaCl) using fine entomological needles under a tereomicroscope, and stored in liquid nitrogen until use. The study was approved by the Animal Care and Use Ethics Committee of Kunming Medical University.

### Peptide purification

**Figure 1:**
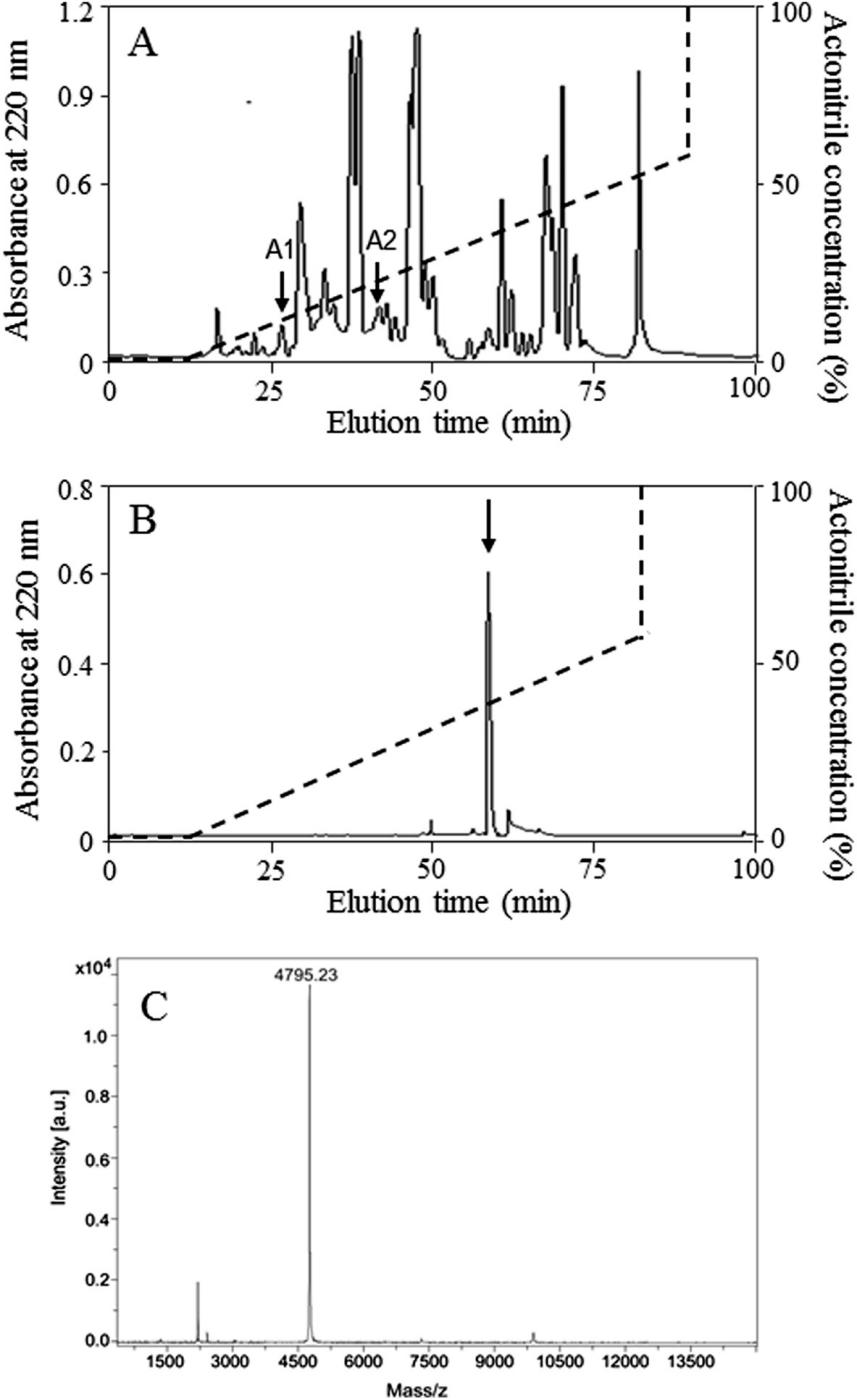
**Isolation of** ***Siba*** **Def from the salivary gland of** ***S. bannaense*** **and MALDI–TOF MS. (A)** The filtrate of the salivary gland homogenate of *S. bannaense* by 10 kDa cut-off was divided by an Inertsil C_4_ RP-HPLC column (25 × 0.46 cm) equilibrated with 0.1% (v/v) trifluoroacetic acid/water. The elution was performed with the indicated gradient of acetonitrile at a flow rate of 1 ml/min. **(B)** The eluted peak of A2 containing antimicrobial activity was further purified by C_18_ RP-HPLC column (25 × 0.46 cm) developed with a linear gradient of 0 to 70% acetonitrile in acidified water at a flow rate of 0.7 ml/min. The purified antimicrobial peptide is indicated by an arrow. **(C)** MALDI-TOF mass spectrometry analysis of the antimicrobial peptide.

1,660 pairs of black fly salivary glands in HEPES saline were thawed and homogenized. After a centrifugation at 12,000 × g for 15 min at 4°C, the supernatant was prepurified through a 10-kDa cut-off Centriprep filter (Millipore, CA). The filtrate was then subjected to RP-HPLC on an Inertsil C_4_ column (25 × 0.46 cm) as illustrated in Figure 1A. The linear gradient elution was performed in a 0–70% acetonitrile containing 0.1% (v/v) trifluoroacetic acid for 80 min. The eluted peaks of A1 and A2 showed antimicrobial activities. The protein peak of A2 was pooled, lyophilized, and further purified by RP-HPLC on a Wondasil C_18_ column (25 × 0.46 cm) as indicated in Figure 1B. Elution was performed with a linear gradient of 0-60% acetonitrile in acidified water over 70 min at a flow rate of 0.7 ml/min. The antimicrobial activity of fractions was determined as indicated below. The interesting eluted peaks were subjected to automated Edman degradation analysis on an Applied Biosystems pulsed liquid-phase sequencer (model ABI 491, USA).

### MALDI-TOF MS analysis

1 μl of the eluted peak with antimicrobial activity was spotted onto a matrix-assisted laser desorption ionization time-of-flight mass spectrometry (MALDI-TOF MS) plate with 1 μl of α-cyano-4-hydroxycinnamic acid matrix (10 mg/ml in 60% acetonitrile) and analyzed by an UltraFlex I mass spectrometer (Bruker Daltonics, Germany) in a positive ion mode.

### cDNA library construction and screening of cDNA encoding defensin

Total RNA was extracted using TRIzol reagent (Invitrogen, USA) from salivary glands of *S. bannaense*. mRNA was purified from the total RNA by affinity chromatography in oligo(dT) cellulose columns (Promega, USA) and then used for cDNA library construction by the In-Fusion SMARTer™ Directional cDNA Library Construction Kit (Takara, Japan) according to the instructions of the manufacturer.

The synthesized second-strand cDNAs was used as a template for PCR to screen the cDNAs encoding defensin. Primers used in this research are shown in Table 1. *Siba*Def-F1 and 3′ PCR primer were used in PCR reaction to screen the 5′ fragments of cDNAs encoding defensin. *Siba*Def-F1 is designed from the amino acid sequence of *Siba*Def determined by Edman degradation, and 3′ PCR primer is based on the adaptor sequence of 3′ In-Fusion SMARTer CDS Primer provided in the kit. The PCR conditions were: 95°C for 5 min and 30 cycles of 95°C (30 s), 58°C (40 s), 72°C (1 min) followed by an extension step at 72°C for 10 min. The PCR product was purified by gel electrophoresis, cloned into pMD19-T vector (Takara, Japan) for sequencing. After the 3′ fragments of cDNA had been obtained, an antisense primer (*Siba*Def-R1) was designed based on the 3′-coding region of defensin cDNA and coupled with 5′ PCR primer provided in the kit to screen the full length cDNA encoding defensin. The PCR conditions were: 95°C for 5 min and 30 cycles of 95°C (30 s), 56°C (30 s), 72°C (50 s) followed by an extension step at 72°C for 8 min. DNA sequencing was performed on an Applied Biosystems DNA sequencer (model ABI PRISM 377, USA).

**Table 1:** **Primer sequences used for cloning and qPCR in this study**

**Primer**	**Sequence (5′ → 3′)**	**Application**
3′ PCR primer	CGGGGTACGATGAGACACCA	3′ end screening
*Siba*Def -F1	TIYTIWSIATHWSNACNCC*	3′ end screening
*Siba*Def -R1	TCGTACATCAGTCAGATCCACCG	5′ end screening
5′ PCR primer	AAGCAGTGGTATCAACGCAGAGT	5′ end screening
*Siba*Def - F2	AGAAGAGCAACCTGCGACCTG	qPCR
*Siba*Def - R2	AGTCAGATCCACCGCCCGAAT	qPCR
*Actin*-F	TGTTGTCACTGTACGCCTCCG	qPCR
*Actin*-R	TGATGTCGCGAACGATTTCCC	qPCR

*Where Y stands for C or T, W stands for A or T, S stands for C or G , H stands for A, C or T, N stands for A, C, G or T, and I stands for hypoxanthine.

### Sequence analysis

Deduced defensin sequence was performed with ExPASy Translate Tool (http://web.expasy.org/translate/). Database searches were performed with Blastx (http://www.ncbi.nlm.nih.gov/), and the amino acid sequence identity between defensin sequences was aligned using ClustalW (http://embnet.vital-it.ch/software/ClustalW.html) [28]. The theoretical isoelectric point (pI) and molecular weight (Mw) were carried out using ExPASy Compute pI/Mw tool (http://web.expasy.org/compute_pi/) [29]. The dendrogram was drawn using the neighbor-joining (NJ) method in the Mega 5 package. A total of 1,000 bootstrap replicates were used to test the reliability of each branch.

### Antimicrobial assay

The microbicidal activity of *Siba*Def was evaluated as described in our previous papers [6,30]. Briefly, bacteria were cultured in Mueller-Hinton broth (MH broth) at 37°C to exponential phase and diluted with fresh MH broth to 5 × 10^5^ colony-forming units (CFUs)/ml. Aliquots (50 μl) of serial dilutions of sample were dispensed into a 96-well microtiter plate and mixed with 50 μl of bacteria inoculums in MH broth. The microtiter plate was incubated at 37°C for 18 h, and the absorbance at 600 nm was measured using an automatic microplate spectrophotometer. The minimal concentrations at which no growth of microorganisms occurred were recorded as minimal inhibitory concentration (MIC).

### Hemolytic assay

Hemolytic assay was conducted as previously reported [31]. Serial dilutions of *Siba*Def were incubated with washed human erythrocytes at 37°C for 30 min and then the cells were centrifuged at 1,000 × g for 5 min. The absorbance of supernatant was measured at 540 nm. 1% (v/v) Triton X-100 was used to determine the maximal hemolysis and 0.9% saline was used as negative control.

### SEM

The morphologic changes of the bacteria treated with *Siba*Def were assessed by SEM as previously reported [31]. Gram-positive bacteria *S. aureus* ATCC 6538 and *B. subtilis* ATCC 6633 were cultured in MH broth to exponential phase respectively, and then incubated with *Siba*Def (1 × MIC) at 37°C for 45 min. After a centrifugation at 1,000 × g for 10 min, bacteria pellets were fixed with 2.5% glutaraldehyde solution for 2 h at 4°C. The bacteria were postfixed in 1% osmium tetroxide for 2 h at 4°C, and dehydrated in a graded series of alcohols. After being mounted onto aluminium stubs and vacuum sputter-coated with gold, the samples were observed with a Hitachi S-4800 SEM under standard operating conditions.

### Bacterial feeding

Bacterial feed experiment was carried out as previously described [32]. The collected *S. bannaense* (200 flies) were fed with 70% sucrose solution *ad libitum.* After starving for 12 hour, black flies were fed through cotton wool with 20% sucrose solution (OD600 = 0.2) containing Gram-positive bacteria *S. aureus* ATCC 6538 or *B. subtilis* ATCC 6633. All the black flies, including the naïve (sugar fed controls), were kept under controlled conditions of temperature (26 ± 2°C), humidity (85-90%), and photoperiod (12 h/12 h). Total RNA was extracted from whole bodies of immune stimulated or naive insects at 12, 24, 36, 48 and 72 h after feeding and processed immediately as described below.

### qPCR

qPCR was performed to analyze the expression of *Siba*Def mRNA in whole bodies of immune stimulated or naive insects, with the housekeeping gene *β-actin* as an endogenous control. As listed in Table 1, primers for *Siba*Def amplification were designed on the *Siba*Def cDNA sequence, and *β-actin* was amplified using primers based on the sequence from black fly *S. vittatum* (GenBank accession number AY083375.1). PrimeScript® Reverse Transcriptase (Takara, Japan) and SYBR green master mix (Takara, Japan) were used following the manufacturer’s instruction.

q-PCR was performed on a Realplex Mastercycler real-time PCR system (Eppendorf, Germany) with the following parameters: 95°C for 2 min, and 40 cycles of 95°C for 30 s,60°C for 30 s. *Siba*Def mRNA expression level was calculated following normalization to *β-actin* by ΔΔCt method. The accuracy of qPCR was verified by melt curve analysis.

### Homology modeling

Defensin homology modeling was performed by Easymodeller version 2.0 [33]. The solution NMR structure of Sapecin (PDB entry code 1L4V) from *Sarcophaga peregrine* (Diptera: Sarcophagidae) was used as the template because this defensin antimicrobial peptide shared the highest identity of 44% with *Siba*Def. The comparative three-dimensional structure model of *Siba*Def was optimized using PYMOL software (http://www.pymol.org).

### Data and statistical analysis

Statistical analyses were performed using GraphPad Prism 5.0 (GraphPad Software Inc., San Diego, CA, USA) and Stata 10.0 software (StataCorporation, College Station, TX, USA). Data were presented as mean ± standard errors of mean, and compared using two-tailed equal variance Student’s *t*-test. P < 0.05 was considered as statistical significance.

## Results

### Characterization of *Siba*Def

**Figure 2:**
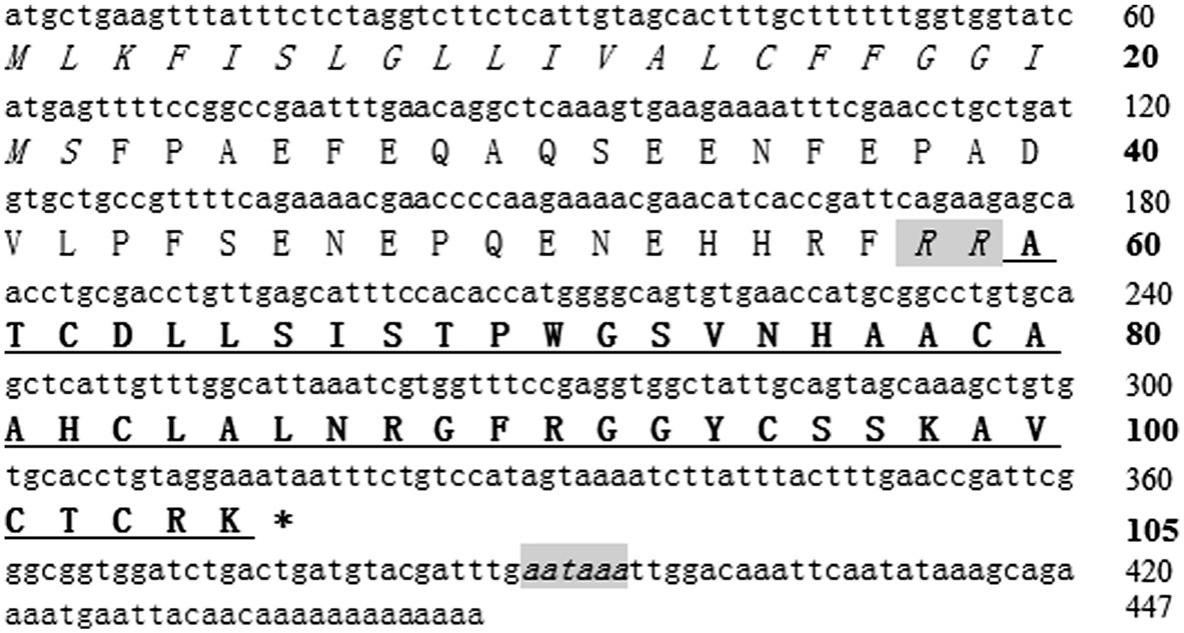
**The cDNA sequence of** ***Siba*** **Def precursor and the deduced amino acid sequence.** Deduced amino acid sequence is shown below the cDNA sequence. The amino acid sequence of mature peptide is underlined and the stop codon is indicated by an asterisk. The dibasic cleavage site and the putative polyadenylation consensus signal are italicized and gray shaded. Amino acid numbers or nucleotide numbers are shown after the sequences.

The fractions with antimicrobial activity (marked by A2) were collected, lyophilized, and further purified by C_18_ RP-HPLC as illustrated in Figure 1B. After Edman degradation, a primary structure of 18 amino acid residues was identified with the following sequence: ATCDLLSISTPWGSVNSA. MALDI-TOF MS analysis (Figure 1C) indicated that the peptide (*Siba*Def) had a measured molecular mass of 4795.23 Da, matching well with the calculated molecular mass 4795.55 Da. The complete nucleotide sequence of cDNA (GenBank accession KJ842485) and deduced amino acid sequence of *Siba*Def precursor are shown in Figure 2. The N-terminal deduced sequence of *Siba*Def precursor is completely consistent with the result of Edman degradation sequencing. The cDNA encoding protein precursor is composed of 105 amino acid residues, including a predicted 22 amino acid signal peptide, a 37 amino acid propeptide region and a 46 amino acid mature *Siba*Def peptide. There is a characteristic dipeptide cleavage site (−R^58^R^59^-) for trypsin-like proteases between propeptide and mature peptide. Analysis using the ExPASy MW/pI tool showed that it has a predicted pI of 8.94. The eluted peak of A1 containing antimicrobial activity was also purified, sequenced and aligned well with other insect cecropins (data not shown).

BLAST search indicated that the amino acid sequence identity between *Siba*Def and their homologues from different insect species varied widely, ranging from 52% to 67%. Surprisingly, *Siba*Def shared the highest identity of 67% (31/46) with the defensin-2 from the human body louse *P. humanus corporis* (Anoplura: Pediculidae).

**Figure 3:**
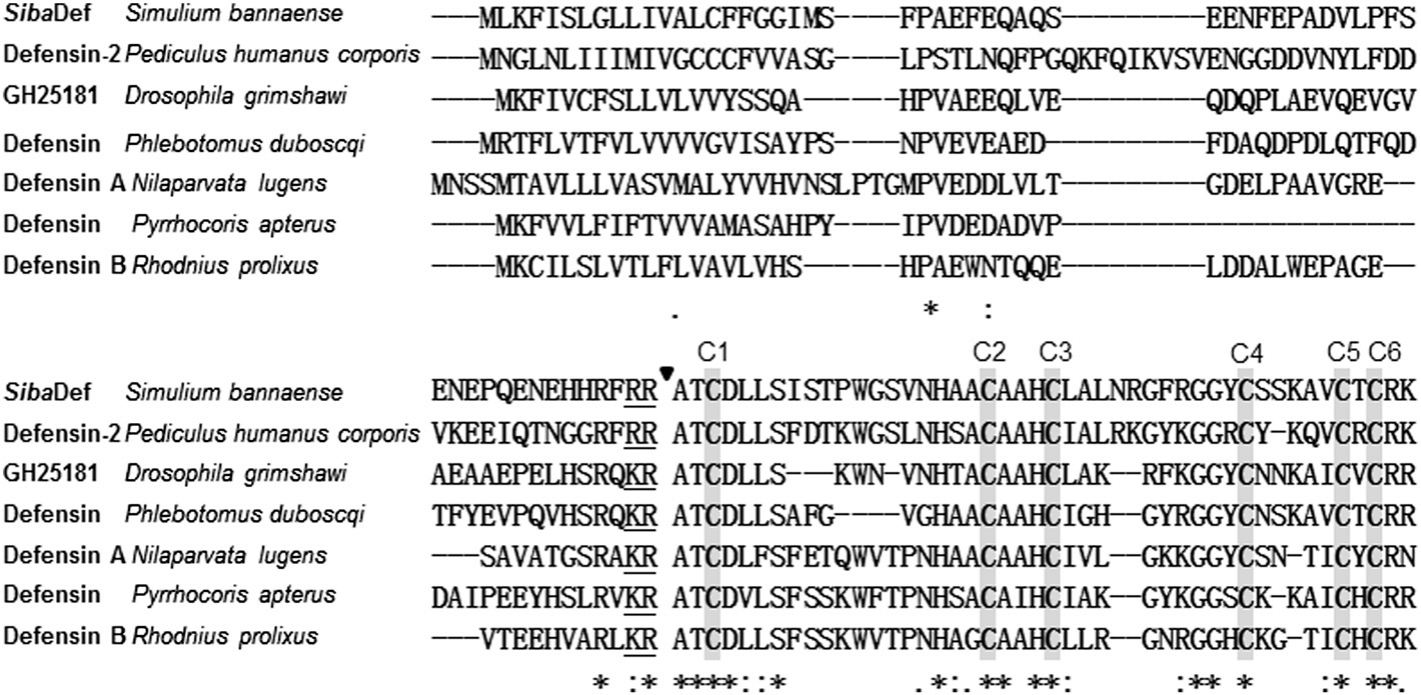
**Alignment of the amino acid sequence of** ***Siba*** **Def with different insect defensins.** These sequences were based on BLAST search results. The symbols under the alignment indicate: (*) identical sites; (:) conserved sites; (.) less conserved sites. The six conserved cysteine residues involved in disulfide bridges are grey shaded and activation peptide cleavage sites are marked with a triangle. GenBank accession numbers for the analyzed sequences are shown in Figure 4.

Multi-sequence alignment of insect defensin precursors (Figure 3) indicated that the signal peptide and propeptide region of these sequences are divergent. However, sixteen amino acids residues within the mature peptides are highly conserved, including a signature motif of six conserved cysteines and an additional ten residues (Ala60, Thr61, Asp63, Ser66, His76, Ala80, His82, Gly92, Gly93 and Arg104). A characteristic feature of all the mature peptides is the presence of an alanine residue and a threonine residue (−AT-) at the N-terminus. In addition, there are two basic residues (−RR- or -RK-) at the C-terminus of the mature peptide, except for the defensin A from *Nilaparvata lugens,* which possesses an arginine residue and an asparagine residue (−RN-) at the C-terminus.

### Phylogenetic analysis

**Figure 4:**
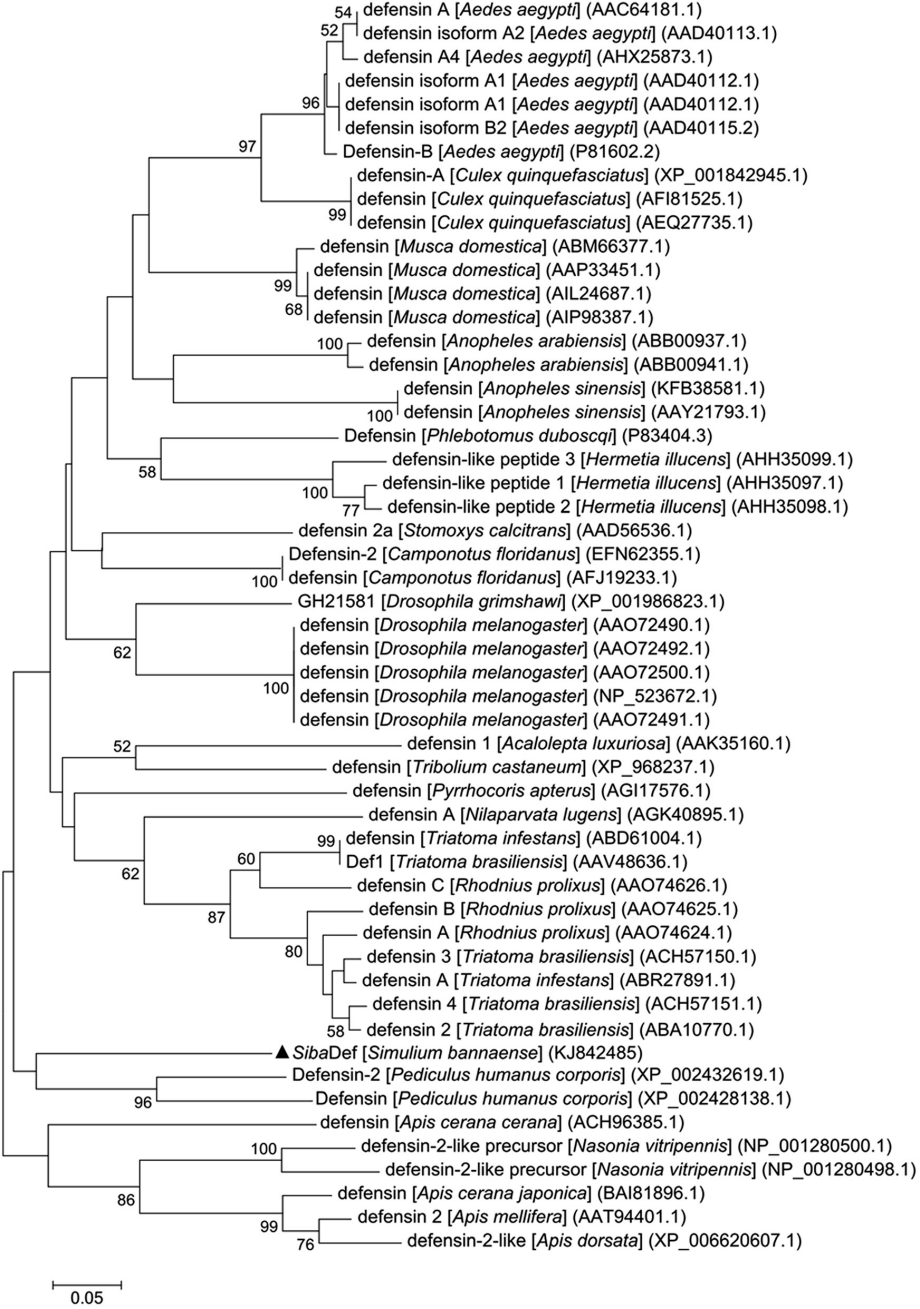
**Phylogenetic tree based on the amino acid sequence of insect defensins.** The numbers on the branches represent the percent bootstrap support and only values over 50% are shown. The bar at the bottom represents 5% amino acid divergence. *Siba*Def is indicated by a triangle.

The phylogenetic tree was generated from 53 defensin-related amino acid sequences (25 insect species including 11 Diptera, 6 Hymenoptera, 4 Hemiptera, 2 Coleoptera, 1 Anoplura and 1 Homoptera). As showed in Figure 4, all defensin sequences are divided into two distinct clusters including 47 sequences derived from different orders of insects (Diptera, Hemiptera, Coleoptera, Anoplura and Homoptera) and 6 sequences derived from hymenopteran insects, respectively. *Siba*Def was grouped together with the anopluran defensins (defensin-2 and defensin) from the human body louse *P. humanus corporis*.

### Antimicrobial activity

The MICs of *Siba*Def against Gram-positive and Gram-negative bacteria were determined. As listed in Table 2, *Siba*Def showed strong antimicrobial activities against four tested Gram-positive bacteria, with MICs ranging from 0.83 μM to 2.29 μM. However, no effect was observed against Gram-negative bacteria *E. coli* and *P. aeruginosa*.

**Table 2:** **Antimicrobial activity of** ***Siba*** **Def**

**Microorganisms**	MIC (μM)*
**Gram-positive bacteria**	
*Staphylococcus aureus* ATCC 6538	0.83
*Bacillus subtilis* ATCC 6633	1.04
*Bacillus cereus* ATCC 14579	2.08
*Micrococcus luteus* ATCC 4698	2.29
**Gram-negative bacteria**	
*Escherichia coli* ATCC 8739	ND
*Pseudomonas aeruginosa* ATCC 9027	ND

*MIC: minimal inhibitory concentration. These MICs represent mean values of three independent experiments performed in duplicates. ND: no detectable activity.

### Hemolysis

Human fresh erythrocytes were used to evaluate the hemolytic activity of *Siba*Def. The result showed *Siba*Def displayed negligible hemolytic activity on human erythrocyte even with peptide concentrations up to 41.71 μM, which is almost 40-fold higher than their corresponding MIC values.

### SEM

**Figure 5:**
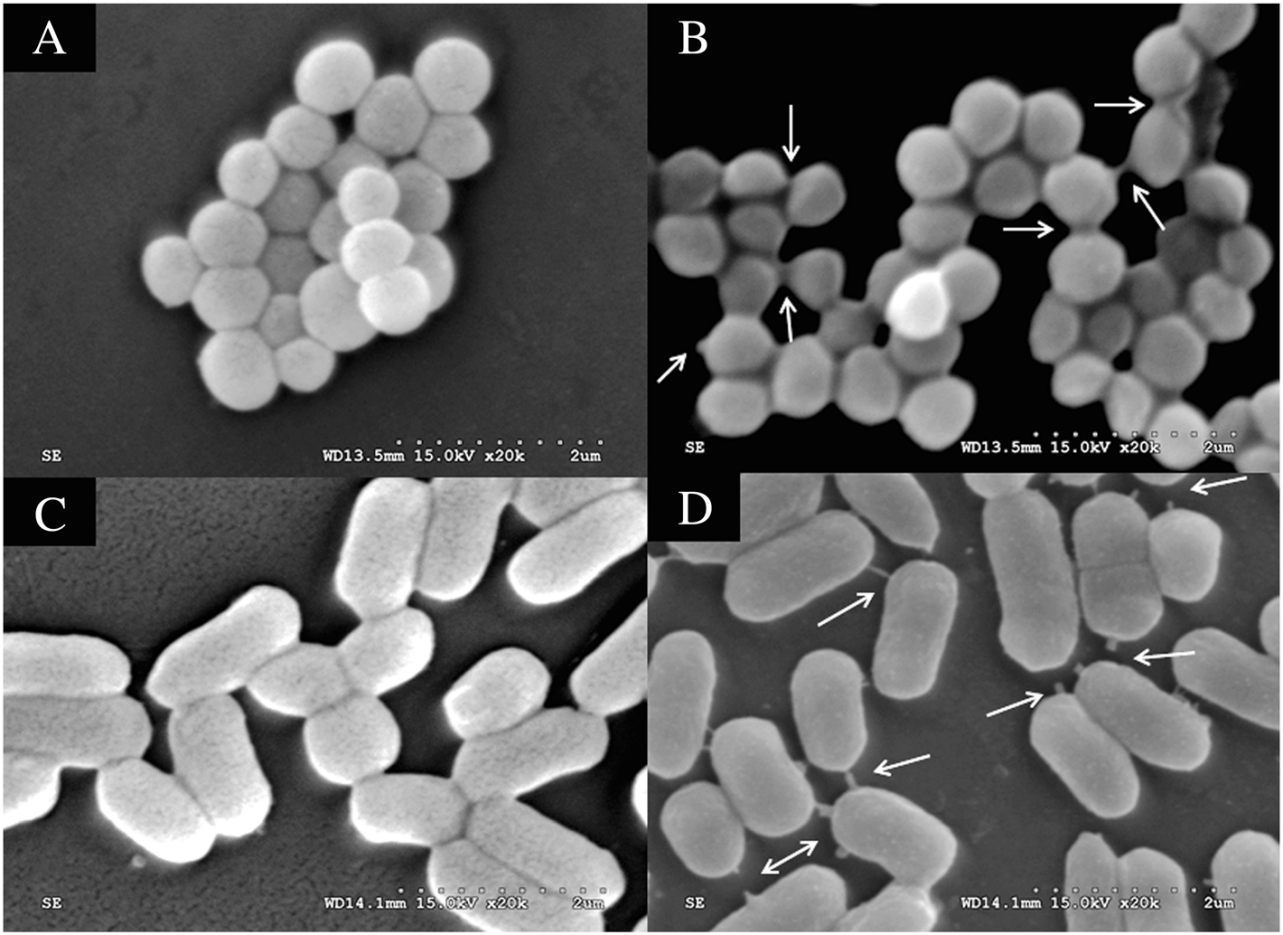
**Scanning electron microscopy analysis of** ***Siba*** **Def-treated bacteria. (A)** Control, untreated *S. aureus*. **(B)** 1 × MIC (0.83 μM) *Siba*Def-treated *S. aureus*. **(C)** Control, untreated *B. subtilis*. **(D)** 1 × MIC (1.04 μM) *Siba*Def-treated *B. subtilis*. Arrow indicates severe leakage of cellular cytoplasmic contents.

SEM was performed to study the possible mechanisms of action of *Siba*Def on Gram-positive bacteria *S. aureus* and *B. subtilis*. In contrast to the untreated *S. aureus* cells (Figure 5A) and *B. subtilis* (Figure 5C), cells treated with *Siba*Def (1 × MIC) showed obvious morphological alterations (Figure 5B, D). The membrane integrity of cells seemed to be disrupted, and there were a large number of filaments on the surface of cells. In addition, exposure of *S. aureus* to *Siba*Def resulted in aggregation (Figure 5B).

### Transcription of *Siba*Def in black flies fed on bacteria

**Figure 6:**
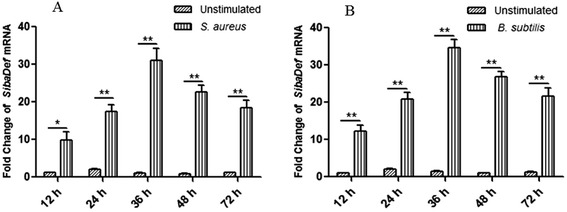
**Fold increase of** ***Siba*** **Def in whole bodies of insects after oral infection with bacteria at different time course. (A)** Fold increase of *siba*Def in insects after *S. aureus* ingestion. **(B)** Fold increase of *Siba*Def in insects after *B. Subtilis* ingestion. Expression levels in whole bodies of bacteria-immunized insects were calculated relative to the level of *Siba*Def in corresponding naive insects, which was arbitrarily defined as 1. Values for infection treatment are significantly different from control values. *P < 0.05, **P < 0.01 significantly different compared to the control (n = 9).

After *S. aureus* or *B. subtilis* ingestion, the expression levels of *Siba*Def mRNA in whole bodies of bacteria-immunized or naive insects were compared at the different time course. As illustrated in Figure 6A, the levels of *Siba*Def mRNA were up-regulated by bacterial-challenge at 12, 24, 36, 48 and 72 h after *S. aureus* ingestion (9.8, 17.4, 31.1, 22.6 and 18.5 fold, respectively). After *B. subtilis* ingestion, the fold increase in defensin transcription at different time course (12.3, 20.9, 34.7, 26.8 and 21.7 fold, respectively) was shown in Figure 6B. The expression of defensin mRNA peaked at 36 h (31.1 and 37.4 fold, respectively) and relatively decreased with time.

### 3D structure analysis of *Siba*Def

**Figure 7:**
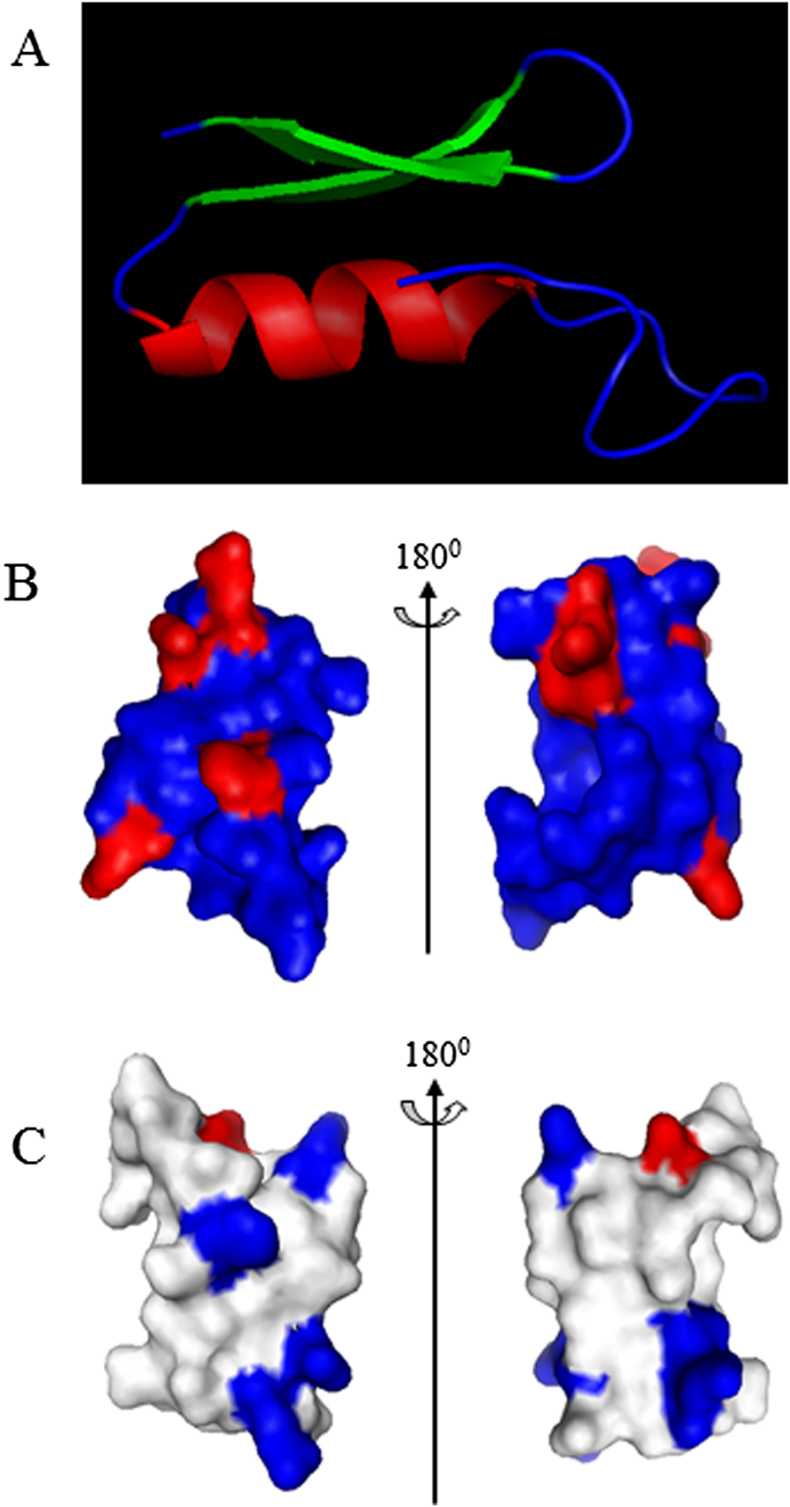
**Homology modeling of** ***Siba*** **Def. (A)** Representation of the homology-derived solution structure of *Siba*Def. It consists of one α-helix (in red), two antiparallel β-sheets (in green) and some random coils (in blue). **(B)** Basic residues (Lys and Arg, in red) are displayed in the structures. **(C)** Electrostatic potential map of *Siba*Def, positively charged region and negatively charged region are shown in blue and red, respectively. Homology model was performed by Easymodeller version2.0 and optimized by using PYMOL software.

The homology modeled structures of *Siba*Def are shown in Figure 7. The common motifs are preserved in the sequence alignment of the template and *Siba*Def structures. It consists of one α-helix (residues Gly13-His23, in red), two antiparallel β-sheets (residues Ala26-Phe31 and Tyr35-Lys39, in green) and some random coils (in blue) locating at both terminal end of *Siba*Def and regions between α-helix and β-sheets (Figure 7A). It also shows the positive charges distribution of *Siba*Def (five basic residues) in the surface of the three-dimensional structure (Figure 7B, in red). Electrostatic surface analysis revealed that several regions of the solution structure surface are positively charged at a neutral pH (Figure 7C, in blue). Taken together, *Siba*Def shared common structural features and electrostatic characteristics with a variety of insect defensins.

## Discussion

Insects lack an acquired immune response, but they have an unspecific cellular response (phagocytosis and encapsulation of invading microorganisms by blood cells) and humoral immune reactions (activation of proteolytic pathways and the rapid synthesis of immune-related peptides) [34]. These peptides are synthesized either by the fat body and various epithelia in holometabolous insects, or by hemocytes in heterometabolous insects [23]. Extensive research in the past decades has established that insect AMPs are ubiquitous and ancient contributors to immune defense against bacterial, fungal and parasitic infections [23,26,35]. As an important blood-sucking insect, there have been comparatively few studies on antimicrobial substances in black fly, especially no defensins have been reported so far.

Here, a novel cationic defensin designated *Siba*Def was purified from the salivary glands of the black fly *S. bannaense*, The structural organization of *Siba*Def precursor (Figure 2) is similar to other insect defensin precursors, comprising a signal peptide sequence, an N-terminal propeptide region containing several aspartic and glutamic acid residues, and the mature peptide at the C-terminus of the precursor. These sequences also share the conserved enzymatic processing sites (−KR- or -RR-) to release the mature peptides. The dibasic cleavage site (Figure 3) has been found in many insect defensins identified from the different orders (Diptera, Anoplura, Coleoptera, Homoptera and Hemiptera) [22,23,26]. The first two amino acid residues (AT) at the N-terminus of *Siba*Def is conserved in phylogenetically higher insects such as mosquitos and triatomines [36]. The consensus motifs of *Siba*Def are C-X_16_-C-X_3_-C-X_11_-C-X_5_-C-X_1_-C (where C is a cysteine, and X is any amino acid except cysteine), which is consistent with the spacing pattern of insect defensins (C-X_5–16_-C-X_3_-C-X_9–11_-C-X_4–7_-C-X_1_-C) [37].

Phylogenetic analysis (Figure 4) showed that *Siba*Def is most closely related to anopluran defensins from the human body louse *P. humanus corporis,* rather than to other dipteran defensins. The evolutionary trends of insect/mosquito defensins have revealed the similar outcomes, in which two dipteran defensins (Agd3 and Agd4) from *Anopheles gambiae* are grouped with lepidopterans more than with mosquitoes. In addition, a lepidopteran defensin (Mbd1) from cabbage moth *Mamestra brassicae* is clustered with the members of the mosquito specific cluster [38]. However, no meaningful explanation for these associations can be found. Previous research on evolution of invertebrate defensins has shown that the available data in hand is inadequate to provide an integrated view of the evolutionary history of AMPs [39]. We suggest that defensins in *P. humanus corporis* and *Siba*Def*,* possibly perform similar functions *in vivo* due to tremendous evolutionary pressure such as the immune pressure imposed by the vertebrate hosts.

Insect defensins are classified into antimicrobial defensins and antifungal defensins according to their *in vitro* activity against bacteria or filamentous fungi [23]. The antimicrobial defensins are known to be active mainly against Gram-positive bacteria at different concentrations (MICs ranging from 0.4 μM to 100 μM) [26,40]. They interact with negatively charged bacterial membranes and insert into membrane bilayers to form pores, leading to membrane permeabilization and disruption [41]. Homology modeling of *Siba*Def (Figure 7) shows that it has a cationic structure with one α-helix and two antiparallel β-strands. The structure contributes to the ability of antimicrobial defensins to kill bacteria [23]. As expected, *Siba*Def shows strong activities (Table 2) against Gram-positive bacteria (MICs ranging from 0.83 to 2.29 μM). SEM analysis indicated that such activities are carried out with a lytic effect on the bacterial membranes (Figure 5). These results confirm that the microbial membrane is a key target for cationic defensins. The potent antimicrobial effect of *Siba*Def facilitates the prevention of bacterial contamination in sugar or blood meal acquisition. However, the actual functions of defensin in the salivary gland of haematophagous insects remain to be elucidated.

The transcript levels of *Siba*Def in whole bodies of insects increase after oral infection with Gram-positive bacteria *S. aureus* or *B. subtilis*, and peak at 36 h post-feeding (Figure 6). In these experiments, black flies challenged with *B. subtilis* express relatively higher levels of defensin mRNA when compared to those insects challenged with *S. aureus* at different time course. Meanwhile, we also observe increased levels of transcription for cecropin in *S. bannaense* after infection (data not shown)*.* These results suggest that *Siba*Def involves in *S. bannaense* innate humoral response and cooperates with other immune-related peptides such as cecropin to control bacterial infection.

## Conclusions

In conclusion, the black fly defensin was first identified in the present work by peptide purification and molecular cloning procedures. This defensin exhibited potent antimicrobial activity against Gram-positive bacteria through the disruption of microbial membrane. Further work needs to be done to investigate what is the actual functions of this immune-related peptide during the meal and bacterial infection.
